# Supplementary material for: Effects of Nordic Walking on Physical Fitness in Patients with Cancer: A Systematic Review
Source: Cancers (Basel). 2025 Sep 29;17(19):3170. doi: 10.3390/cancers17193170 (PMC12524311; doi:10.3390/cancers17193170)
Supplement: Supplementary file 1 [file cancers-17-03170-s001.zip › cancers-3878613-supplementary.pdf]

SUPPLEMENTARY MATERIALS

Supplementary S1: Search strategies

Pubmed

1.	“Nordic Walking”[Mesh]	38
2.	“Nordic Walking”[Title/Abstract]	374
3.	1 OR 2	380
4.	“Neoplasms”[Mesh]	4,036,496
5.	“Cancer”[Title/Abstract]	2,364,081
6.	"Oncolog*"[Title/Abstract]	238,451
7.	4 OR 5 OR 6	4,764,446
8.	Randomizedcontrolledtrial[Filter]	626,217
9.	Random*[Title/Abstract]	1.571.715
10.	7 OR 8	7,702,946
11.	3 AND 6 AND 10	12

Cochrane Library

#1	MeSH descriptor: [Nordic Walking] explode all trees	13
#2	("nordic walking"):ti,ab,kw	330
#3	#1 OR #2	330
#4	MeSH descriptor: [Neoplasms] explode all trees	127536
#5	(cancer):ti,ab,kw	214935
#6	(oncolog*):ti,ab,kw	35156
#7	#4 OR #5 OR #6	260761
#8	#3 AND #7	33 (33 Trials)

Web of Science

1. TI=("nordic walking")	329
2. AB=("nordic walking")	392
3. #2 OR #1	474
4. TI=(neoplasm*)	42.923
5. AB=(neoplasm*)	119.122
6. TI=(Oncolog*)	91,218
7. AB=(Oncolog*)	179,782
8. TI=(cancer)	1,766,586
9. AB=(cancer)	2,013,021
10. #4 OR #5 OR #6 OR #7 OR #8 OR #9	3,008,678
11. TI=(random*)	580.049

12. AB=(random*)	2.203.299
13. #10 OR #11	2,388.784
14. DT=(Article)	56,255,481
15. #3 AND #10 AND #13 AND #14	8

Pedro
Abstract and Title: “Nordic Walking”
Subdiscipline: Oncology
Method: Clinical trial

7 results

Supplementary S2: Details of excluded articles with reasons

Wrong publication type (conference presentation/poster) (n = 6)

1. Bucciarelli V, Bianco F, Di Blasio A, Morano T, Izzicupo P, Napolitano G, et al. The role of physical exercise on endothelial dysfunction and metabolic improvement in women after breast-cancer surgery: a pilot study. European heart journal [Internet]. 2017;38:124. Available from: https://www.cochranelibrary.com/central/doi/10.1002/central/CN-01469015/full

2. Bucciarelli V, Bianco F, Di Blasio A, Morano T, Tuosto D, Mucedola F, et al. The differential effects of a short term aerobic or resistance physical exercise protocol in the improvement of endothelial function and cardiovascular efficiency in women after breast-cancer surgery. European heart journal cardiovascular Imaging [Internet]. 2017;18:iii24. Available from: https://www.cochranelibrary.com/central/doi/10.1002/central/CN-01452115/full

3. Bucciarelli V, Bianco F, Di Blasio A, Morano T, Tuosto D, Mucedola F, et al. Ventricular-arterial coupling and acute levels of DHEA-S in breast cancer survivors after a short term physical exercise protocol. European heart journal cardiovascular Imaging [Internet]. 2019;20:i50. Available from: https://www.cochranelibrary.com/central/doi/10.1002/central/CN-02085610/full

4. Bucciarelli V, Bianco F, Di Blasio A, Morano T, Tuosto D, Mucedola F, et al. The effects of adherence to physical exercise on cardiovascular efficiency in breast cancer survivors. European heart journal, supplement [Internet]. 2019;21:J39. Available from: https://www.cochranelibrary.com/central/doi/10.1002/central/CN-02326971/full

5. Fields J, Richardson A, Fenlon D. Nordic walking as a physical activity intervention for aromatase inhibitor associated arthralgia: a feasibility study. European journal of surgical oncology [Internet]. 2015;41(6):S76-S77. Available from: https://www.cochranelibrary.com/central/doi/10.1002/central/CN-01172523/full

6. Jastrzębski D, Rutkowska A, Rutkowski S, Kostorz S, Zebrowska A, Ziora D, et al. Short-time exercise-induced rehabilitation in non-small cell lung cancer patients during in-hospital chemotherapy treatment: a randomized controlled trial. European Respiratory Journal [Internet]. 2017 Dec 6 [cited 2025 Jun 3];50(suppl 61). Available from: https://publications.ersnet.org/content/erj/50/suppl_61/OA4671

Wrong intervention (n = 1)

1. Rutkowska A, Jastrzebski D, Rutkowski S, Zebrowska A, Stanula A, Szczegielniak J, et al. Exercise Training in Patients With Non-Small Cell Lung Cancer During In-Hospital

Chemotherapy Treatment A RANDOMIZED CONTROLLED TRIAL. JOURNAL OF CARDIOPULMONARY REHABILITATION AND PREVENTION. 2019;39(2):127–33.

Wrong outcome (n = 4)

1. Czerwinska-Ledwig O, Jurczyszyn A, Piotrowska A, Pilch W, Antosiewicz J, Zychowska M. The Effect of a Six-Week Nordic Walking Training Cycle on Oxidative Damage of Macromolecules and Iron Metabolism in Older Patients with Multiple Myeloma in Remission-Randomized Clinical Trial. International journal of molecular sciences [Internet]. 2023;24(20). Available from: https://www.cochranelibrary.com/central/doi/10.1002/central/CN-02608694/full

2. di Blasio A; Morano T; Bucci I; di Santo S; d'Arielli A; Castro CG; Cugusi L; Cianchetti E; Napol... Physical exercises for breast cancer survivors: effects of 10 weeks of training on upper limb circumferences. Journal of Physical Therapy Science 2016 Oct;28(10):2778-2784. 2016;

3. Czerwinska-Ledwig O, Vesole D, Piotrowska A, Gradek J, Pilch W, Jurczyszyn A. Effect of a 6-Week Cycle of Nordic Walking Training on Vitamin 25(OH)D3, Calcium-Phosphate Metabolism and Muscle Damage in Multiple Myeloma Patients–Randomized Controlled Trial. Journal of clinical medicine [Internet]. 2022;11(21). Available from: https://www.cochranelibrary.com/central/doi/10.1002/central/CN-02496173/full

4. Di Blasio A, Morano T, Napolitano G, Bucci I, Di Santo S, Gallina S, et al. Nordic Walking and the Isa Method for Breast Cancer Survivors: Effects on Upper Limb Circumferences and Total Body Extracellular Water - a Pilot Study. BREAST CARE. 2016;11(6):428–31.

Duplicate (n = 1)

1. Hanuszkiewicz J, Woźniewski M, Malicka I. The Influence of Nordic Walking on Isokinetic Trunk Muscle Endurance and Sagittal Spinal Curvatures in Women after Breast Cancer Treatment: Age-Specific Indicators. Int J Environ Res Public Health. 2021 Mar 2;18(5):2409. doi: 10.3390/ijerph18052409. PMID: 33801189; PMCID: PMC7967775.

Supplementary S3: Fixed effects model analysis

a. Cardiorespiratory endurance post-intervention

Study or Subgroup	Nordic Walking Mean	Nordic Walking SD	Nordic Walking Total	No intervention Mean	No intervention SD	No intervention Total	Weight	Mean Difference IV, Fixed, 95% CI
Casanovas-Álvarez A, 2024 [18]	638.87	68.43	30	514.81	123.01	31	90.8%	124.06 [74.31, 173.81]
Cunningham E, 2020 [19]	512.3	153	4	523.6	46	4	9.2%	-11.30 [-167.87, 145.27]
**Total (95% CI)**			**34**			**35**	**100.0%**	**111.65 [64.24, 159.06]**

Heterogeneity: Chi² = 2.61, df = 1 (P = 0.11); I² = 62%
Test for overall effect: Z = 4.62 (P < 0.00001)

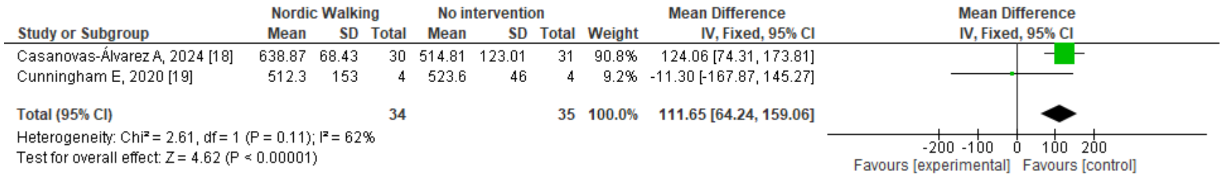

b. Muscle strength post-intervention

Study or Subgroup	Nordic Walking Mean	Nordic Walking SD	Nordic Walking Total	No intervention Mean	No intervention SD	No intervention Total	Weight	Std. Mean Difference IV, Fixed, 95% CI
**1.2.1 Hand grip**								
Casanovas-Álvarez A, 2024 [18]	23.1	5.1	32	21.1	6.01	32	42.7%	0.35 [-0.14, 0.85]
Cunningham E, 2020 [19]	31.3	13.7	4	22.75	4.17	4	4.8%	0.73 [-0.74, 2.21]
**Subtotal (95% CI)**			**36**			**36**	**47.5%**	**0.39 [-0.08, 0.86]**
Heterogeneity: Chi² = 0.23, df = 1 (P = 0.63); I² = 0%								
Test for overall effect: Z = 1.64 (P = 0.10)								
**1.2.2 Upper extremity**								
Malicka I, 2021 [22]	161.17	49.2	23	155.14	48.7	15	24.6%	0.12 [-0.53, 0.77]
**Subtotal (95% CI)**			**23**			**15**	**24.6%**	**0.12 [-0.53, 0.77]**
Heterogeneity: Not applicable								
Test for overall effect: Z = 0.36 (P = 0.72)								
**1.2.3 Trunk**								
Hanuszkiewicz JM, 2020 [21]	483.02	315.94	19	234.05	206.48	20	23.7%	0.92 [0.26, 1.58]
**Subtotal (95% CI)**			**19**			**20**	**23.7%**	**0.92 [0.26, 1.58]**
Heterogeneity: Not applicable								
Test for overall effect: Z = 2.71 (P = 0.007)								
**1.2.4 Lower body**								
Cunningham E, 2020 [19]	14.3	4.2	3	11.8	3.2	4	4.3%	0.58 [-0.99, 2.15]
**Subtotal (95% CI)**			**3**			**4**	**4.3%**	**0.58 [-0.99, 2.15]**
Heterogeneity: Not applicable								
Test for overall effect: Z = 0.73 (P = 0.47)								
**Total (95% CI)**			**81**			**75**	**100.0%**	**0.46 [0.14, 0.78]**

Heterogeneity: Chi² = 3.21, df = 4 (P = 0.52); I² = 0%
Test for overall effect: Z = 2.78 (P = 0.005)
Test for subgroup differences: Chi² = 2.98, df = 3 (P = 0.39), I² = 0%

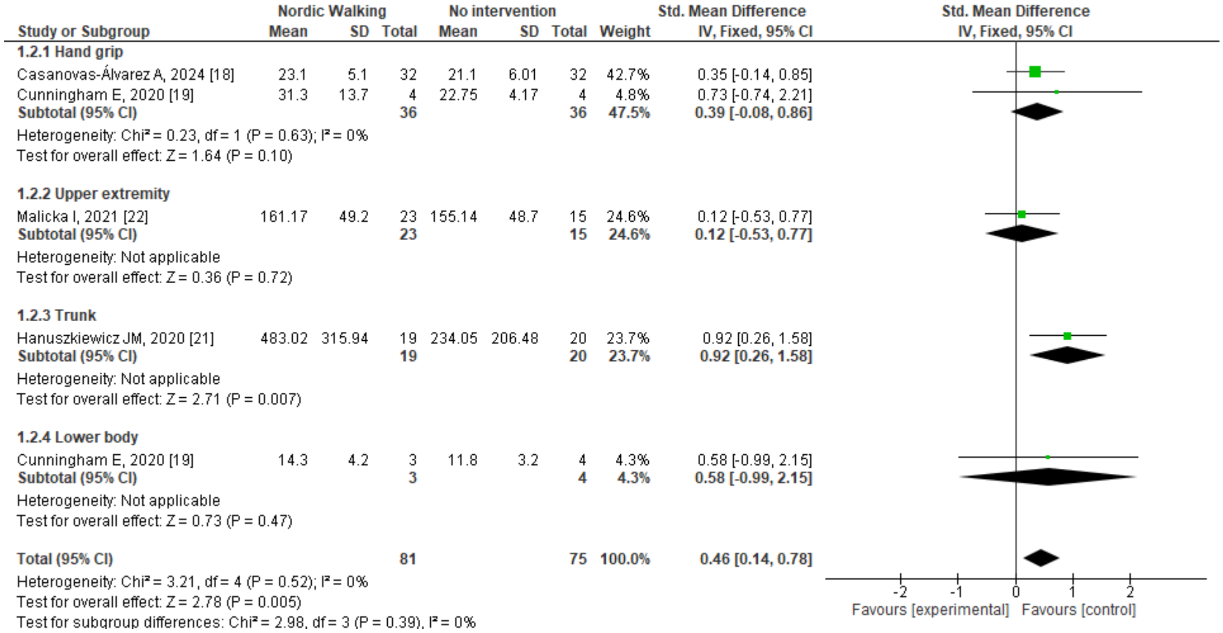

c. Physical activity level

Mean difference

Study or Subgroup	Nordic Walking Mean	Nordic Walking SD	Nordic Walking Total	No intervention Mean	No intervention SD	No intervention Total	Weight	Mean Difference IV, Fixed, 95% CI
Casanovas-Álvarez A, 2024 [18]	4,301	3,015	30	1,137	980	31	93.7%	3164.00 [2031.30, 4296.70]
Cunningham E, 2020 [19]	5,051	3,455.7	3	1,743.3	1,987	4	6.3%	3307.70 [-1060.72, 7676.12]
Fields J, 2016 [20]	0	0	0	0	0	0		Not estimable
**Total (95% CI)**			**33**			**35**	**100.0%**	**3173.05 [2076.61, 4269.49]**

Heterogeneity: Chi² = 0.00, df = 1 (P = 0.95); I² = 0%
Test for overall effect: Z = 5.67 (P < 0.00001)

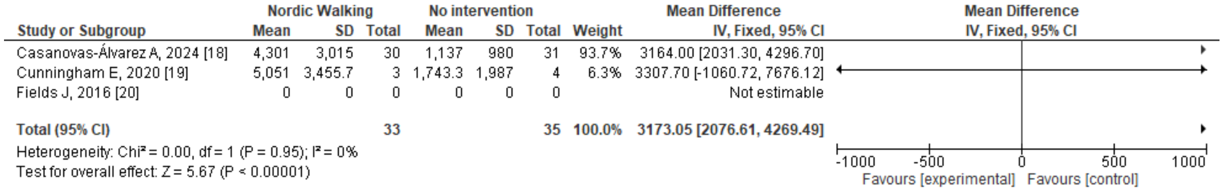

Standardized mean difference

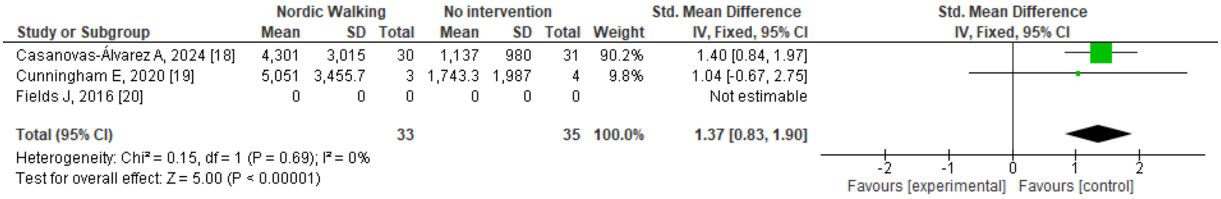

Supplementary S4: Sensitivity analysis

Muscle strength post-intervention

Study excluded	Result (Std. MD and 95%CI)
Casanovas-Alvarez, 2024	0.54 [0.11, 0.96]
Cunningham, 2020	0.45 [0.11, 0.78]
Malicka, 2021	0.57 [0.20, 0.94]
Hanuszkiewicz, 2020	0.32 [-0.05, 0.68]
Cunningham, 2020	0.46 [0.11, 0.80]

Supplementary S5: Certain of the evidence

Certainty assessment							№ of patients		Effect		Certainty	Importance
№ of studies	Study design	Risk of bias	Inconsistency	Indirectness	Imprecision	Other considerations	Nordic Walking	No intervention	Relative (95% CI)	Absolute (95% CI)		
**Cardiorespiratory endurance**												
2	randomised trials	serious[a]	serious[b]	not serious	serious[c]	none	33	35	-	MD **84.78 higher** (35.63 lower to 205.19 higher)	⊕◯◯◯ Very low[a,b,c]	CRITICAL
**Muscle strength**												
4	randomised trials	serious[d]	not serious	not serious	serious[c]	none	81	75	-	SMD **0.46 higher** (0.14 higher to 0.78 higher)	⊕⊕◯◯ Low[c,d]	CRITICAL
**Physical activity level**												
2	randomised trials	serious[e]	not serious	not serious	not serious	none	33	35	-	MD **3181.51 higher** (2085.08 higher to 4277.93 higher)	⊕⊕⊕◯ Moderate[e]	IMPORTANT

CI: confidence interval; **MD:** mean difference; **SMD:** standardised mean difference

Explanations

a. Lack of assessor blinding may have introduced detection bias, as the 6-minute walk test is susceptible to influence from verbal encouragement or variations in test administration

b. Downgraded due to variation in effect estimates depending on the statistical model used (fixed-effect vs. random-effects)

c. Downgraded due to wide confidence intervals

d. Lack of assessor blinding may have introduced detection bias

e. Downgraded due to use of self-reported physical activity (IPAQ) without participant blinding, increasing the risk of response and performance bias.
